# Comment on "*Hotair* Is Dispensable for Mouse Development"

**DOI:** 10.1371/journal.pgen.1006406

**Published:** 2016-12-15

**Authors:** Lingjie Li, Jill A. Helms, Howard Y. Chang

**Affiliations:** 1 Program in Epithelial Biology Stanford University School of Medicine, Stanford, California, United States of America; 2 Department of Surgery, Stanford University School of Medicine, Stanford, California, United States of America; University of California San Francisco, UNITED STATES

Amandio et al. [1] reanalyzed a *Hotair* knockout (KO) mouse allele previously reported by Li et al. [2]. The authors evaluated the phenotypes in different genetic backgrounds, performed transcriptome profiling of cells derived from different tissues and developmental stages, and, based on such investigations, highlighted different potential regulatory mechanisms. (e.g., cis versus trans effects).

Li et al. analyzed *Hotair* KO on the C57BL/6 background and examined the lumbar and wrist skeletons. These phenotypes were examined because prior work ([3], also by Duboule lab) showed that a *HoxD* locus transgene caused lumbosacral homeotic transformation (lumbar 6 transformed to sacral 1, L6→S1, also termed L5 based on the last lumbar segment). In C57BL/6 background, Li et al. found 6% of wild-type (WT) animals had L5, whereas 58% of the *Hotair* KO mice have L5, consistent with an L6→S1 transformation [2]. Amandio et al. analyzed the *Hotair* KO in a mixed CBAxBL6 background. In this background, approximately 80% of WT animals already had L5, and there was no additional lumbar skeletal phenotype (e.g., L4) in the KO, which may not be surprising, as the Hotair expression domain is located more posteriorly. Amandio et al. also found lower frequency of a caudal skeletal transformation (C4) than reported by Li et al. and independently by Lai et al. in two *Hotair-LacZ* KO alleles [2,4]. Amandio et al. did not observe malformation of wrist bones reported by Li et al., although Li et al. performed histologic sections at multiple stages that may be more sensitive than whole mounts at one time point done in [1]. Genetic background has a profound influence on KO phenotypes [5], and this lesson extends to long noncoding RNAs (lncRNAs) [6].

Li et al. analyzed RNA expression and histone modification patterns from tail tip fibroblasts (TTF) obtained from newborn mice [2]. TTF were chosen because (i) they represented a relatively homogenous cell type with defined positional identity, (ii) single cell analysis showed most TTF cells expressed Hotair, and (iii) TTF allowed comparison with human fibroblasts in which human HOTAIR was characterized. Li et al. found derepression of *HoxD* and several imprinted genes in the KO. In contrast, Amandio et al. performed RNA-seq on dissected E12.5 embryo fragments and whole mount in situ hybridization on E12.5 embryos and did not detect changes in HoxD expression [1]. Each embryo fragment is expected to contain a variety of different cell types. Indeed, Amandio et al. identify hundreds of genes with apparent differences in expression between WT and KO in the CBAxBL6 background, and the largest gene derepression was observed in posterior trunk and genital tubercle, where Hotair expression is the highest in WT tissues. However, many gene expression differences were identified between WT and KO even in tissues that do not express Hotair; thus, it is unclear if such differences reflect direct effects of Hotair or technical variations from microdissection. Nonetheless, this information expands the potential genes affected in vivo, directly or indirectly, by *Hotair* deletion.

*Hotair* is located in the *HoxC* locus between *Hoxc11* and *Hoxc12*. Amandio et al. focused on the *HoxC* locus, observed alterations in *Hoxc11* expression domain, and discovered new transcribed regions at the junction of the *Hotair* deletion [1]. These cis effects should be taken into consideration in future studies and highlight the interweaved nature of regulatory elements and pervasive transcription in the *Hox* loci and in mammalian genomes. Future studies using ectopic *Hotair* transgene may be able to separate cis versus trans effects of the lncRNA. Additionally, focal promoter deletion or insertion of polyadenylation sequence to truncate lncRNA in a strand-specific manner may better target lncRNA transcription while minimally affecting its genomic locus in cis [7,8].
